# Surface NKG2C Identifies Differentiated αβT-Cell Clones Expanded in Peripheral Blood

**DOI:** 10.3389/fimmu.2020.613882

**Published:** 2021-02-16

**Authors:** Elena I. Kovalenko, Ivan V. Zvyagin, Maria A. Streltsova, Artem I. Mikelov, Sofya A. Erokhina, William G. Telford, Alexander M. Sapozhnikov, Yury B. Lebedev

**Affiliations:** ^1^ Department of Immunology, Shemyakin-Ovchinnikov Institute of Bioorganic Chemistry, Moscow, Russia; ^2^ Department of Genomics of Adaptive Immunity, Shemyakin-Ovchinnikov Institute of Bioorganic Chemistry, Moscow, Russia; ^3^ Center of Life Sciences, Skolkovo Institute of Science and Technology, Moscow, Russia; ^4^ Experimental Transplantation and Immunology Branch, National Cancer Institute, National Institutes of Health, Bethesda, MD, United States

**Keywords:** TCR repertoire, NKT-like cells, NKG2C, T cell differentiation, cytomegalovirus infection

## Abstract

T cells that express CD56 in peripheral blood of healthy humans represent a heterogeneous and poorly studied subset. In this work, we analyzed this subset for NKG2C expression. In both CD56^+^ and CD56^−^ subsets most of the NKG2C^+^ T cells had a phenotype of highly differentiated CD8^+^ TEMRA cells. The CD56^+^NKG2C^+^ T cells also expressed a number of NK cell receptors, such as NKG2D, CD16, KIR2DL2/DL3, and maturation marker CD57 more often than the CD56^−^NKG2C^+^CD3^+^ cells. TCR β-chain repertoire of the CD3^+^CD56^+^NKG2C^+^ cell fraction was limited by the prevalence of one or several clonotypes which can be found within the most abundant clonotypes in total or CD8^+^ T cell fraction TCRβ repertoire. Thus, NKG2C expression in highly differentiated CD56^+^ T cells was associated with the most expanded αβ T cell clones. NKG2C^+^ T cells produced almost no IFN-γ in response to stimulation with HCMV pp65-derived peptides. This may be partially due to the high content of CD45RA^+^CD57^+^ cells in the fraction. CD3^+^NKG2C^+^ cells showed signs of activation, and the frequency of this T-cell subset in HCMV-positive individuals was positively correlated with the frequency of NKG2C^+^ NK cells that may imply a coordinated in a certain extent development of the NKG2C^+^ T and NK cell subsets under HCMV infection.

## Introduction

Memory T cells play an important role in the immune response to infection or tumor. Differentiation of T cells triggered by encounters with antigen leads to the formation of a set of central memory and effector memory cells, including highly differentiated effector memory cells re‐expressing CD45RA (TEMRA) cells characterized in humans by a CD45RA^+^CD45RO^−^CCR7^−^CD28^−^CD27^−^ phenotype. Memory T cells differ in homing potential and effector functions and provide a more intense and specialized response to the pathogen (1–3). Some of the T cells circulating in adults express surface molecules normally expressed by NK cells. KIR receptors, CD94/NKG2 heterodimers and NKG2D receptor are found on the surface of αβ T cells in peripheral blood. The percentage of T cells expressing these receptors in total αβ T cell fraction varies from <1% to 15% and may be even higher in total γδ T cells (4). Typically, NK cell receptors are expressed on memory T cells as evidenced in particular by a positive correlation between the proportion of CD28^−^ T cells and the proportion of T cells expressing NK cell receptors (5). These CD28-negative T cells express granzyme B, perforin and FasL (6, 7). They also express CD57 antigen, which marks the terminal stage of T cell and NK cell differentiation (8).

One molecule characteristic of NK cells is the neuronal cell adhesion molecule NCAM/CD56. Expression of this marker on T cells makes it possible to isolate a subset of so-called NKT-like cells, named so as to distinguish them from the conventional Vα24-Jα18^+^Vb11^+^ (6b11^+^) invariant NKT cells capable of reacting with α-galactosylceramide (9, 10). These cells are also called NK-like T cells or simply CD56^+^ T cells. The CD56^+^ subset can range from 4 to 30% of all T cells. It is rather heterogeneous, characterized by high intracellular level of granzyme B and perforin and cytolytic capacity (11). Another characteristic of these cells is a reduced ability to divide and an increased resistance to apoptosis (12, 13). CD56^+^ T cells have shorter telomeres and have a lower level of beta-chain variable region complexity (TREC copy number) than CD56^−^ cells, suggesting that they underwent intense post-thymic proliferation (13). Although this subset usually contains a significant number of CD4^+^ cells, CD8^+^ cells can predominate (12). These unique CD8^+^ effectors are capable of mediating TCR-independent CD56-mediated immune cascades (12). CD56^+^ T cell subset increase in number in old age as well as in many chronic inflammatory diseases (10, 14). CD8^+^CD56^+^ T cells can be obtained by cytokine stimulation, producing cytokine-induced cells usable for immunotherapy (15). For example, IL-15 induces formation of cytolytic cell population with effector memory phenotype (16).

There are some data on the distribution of NK cell receptors in the CD56^+^ T cell subset. KIR^+^ T cell residency shown for CD56^+^ compartment has been fairly well studied (13). Although KIR expression can be found in a small portion of CD4^+^ cells, the most of KIR^+^ T cells are CD8^+^. It was noted that KIR^+^CD8^+^ T cells can be isolated from peripheral blood of normal adult individuals (17). The size of the KIR^+^CD8^+^ T cell subset increases with age, and comprises up to 30% of CD8^+^ T cells in elderly individuals. It is noted that most of these cells have a memory cell phenotype; they lack CCR7 expression, and most of them do not express costimulatory molecules CD28 and CD27 (17).

A large number of CD3^+^CD56^+^ cells are found in patients with coronary artery disease (18), lung cancer (19). During viral infections, the content of CD56^+^ T cells can both increase, as with HIV-1 infection and chronic hepatitis B (20, 21), or decrease as with viral hepatitis E (22). Increased numbers of CD56^+^ T cells were also observed in healthy individuals infected with cytomegalovirus (HCMV), one of the most common viruses in the human population (23). It has been shown that this infection is accompanied by T cell expansion with the accumulation of highly differentiated CD8^+^ cells and a decrease in the pool of naive T cells (24). Infection of the human population with HCMV in different countries reaches 50% to 100%. Effective control of this evolutionarily ancient viral infection requires the involvement of both innate and adaptive immunity with a central role for both T lymphocytes and NK cells. HCMV-specific T cells typically respond to the pp65 and IE1 peptides (25). It has been found that in seropositive people the proportion and absolute number of CD8^+^CD57^+^ T cells is higher in all age groups than in seronegative people (8). The accumulation of memory T cells with age is called CD8^+^ T inflation (26, 27). According to Chan et al., CD56^+^ T cells during HCMV infection contain significantly more KIR^+^ cells than T cells from seronegative donors (13). These cells begin to actively proliferate upon reactivation of HCMV in bone marrow transplant recipients, and can lyse targets of NK cells upon activation with cytokines. At the same time, the heterogeneous subset of highly differentiated T cells expressing CD56 is still not fully characterized. It remains unclear whether the differentially described T cells from the numerous studies belong to the same subset.

CD8^+^ T cells can express the NKG2C receptor, which recognizes non-classical major histocompatibility complex molecule HLA-E. In some individuals, NKG2C can also be found on a subset of gamma delta T cells, or even certain CD4^+^ cells (28, 29). In NK cells, this receptor, as a part of heterodimer with CD94, induces an activating signal. The emergence and persistence of the NKG2C-expressing NK cell pool is associated with the development of HCMV infection, along with the presence of pro-inflammatory cytokines such as IL-12 (30). Upon cell infection, the cytomegalovirus UL40 protein induces an increase in the surface expression of HLA-E that occurs independently of TAP (31). This phenomenon is considered to be a mechanism for virus escape from the immune surveillance carried out by NK cells, due to the interaction of HLA-E with the inhibitory receptor NKG2A/CD94 (32). Still, HLA-E–bound peptides have the essential role in selective NKG2C-mediated recognition and activation of NK cells (33, 34). The HLA-E molecule can present only a limited set of peptides, mainly peptides from the leader sequences of other MHC-I molecules. It has been shown that some peptides of the UL40 protein from certain HCMV strains have homology with these HLA-I–derived peptides and can also be presented by HLA-E and cause activation of NKG2C-positive NK cells, in particular, increasing their antibody-dependent response (35, 36). CD8^+^ T cells expressing KIR were also shown to bind HLA-E tetramers loaded with a peptide derived from UL40 (37). It is believed that such T cells recognize peptides in the context of HLA-E *via* the T cell receptor (38). It is assumed that genetic factors in conjunction with the HCMV strain are essential for the emergence of this fraction. However, there remains a lot of uncertainty about how the adaptive and innate immunity, represented by cytotoxic T lymphocytes and NK cells, interact in response to cytomegalovirus infection.

In our work, we characterized highly differentiated T cells expressing the NKG2C receptor. We have shown that the majority of NKG2C^+^ cells in the CD56^+^ T cell subset belongs to one or several CD8^+^ T cell clones, while often constituting a significant part of the total population of CD8^+^ αβ T cells. Most of the NKG2C^+^ T cells expressed other receptors typical of NK cells, such as NKG2D, KIR, and CD16, and were positive for CD57. NKG2C^+^ T cells often demonstrated signs of activation, and especially NKG2C^+^CD56^−^ subset. The proportions of NKG2C^+^ T cells and NKG2C^+^ NK cells were positively correlated, which indirectly indicates the coordinated formation of these subsets. Interestingly, the expanded NKG2C^+^ T cell clone was also found in a HCMV-negative donor. Highly differentiated NKG2C-positive T cells did not exhibit HCMV-specific IFN-γ response, but showed increased natural and antibody-dependent cellular cytotoxicity characteristic of NK cells.

## Materials and Methods

### Subjects

Healthy individuals participated in the study conducted in accordance with the recommendations of the ethics committee of Pirogov Russian National Research Medical University (protocol #169 from 20.11.2017). The volunteers gave their informed consent prior to the study. HCMV-specific IgG antibodies were indentified in the serum samples of volunteers using a commercially available ELISA kit (Vector-Best, Russia) according to the manufacturer’s protocol.

### Isolation of Peripheral Mononuclear Cells

Peripheral blood mononuclear cells (PBMC) were obtained by gradient centrifugation using a standard Ficoll solution (PanEco, Russia), density 1.077. For some experiments, CD8^+^ and CD4^+^ T cells were isolated by magnetic immunoseparation, according to the manufacturer’s protocol (CD8^+^ T Cell Isolation Kit, human, and CD4+ T Cell Isolation Kit, human; Miltenyi Biotec, Germany).

### Cell Lines

The K562 and C1R cell lines (ATCC, USA) were cultivated in RPMI-1640 medium (PanEco, Russia) supplemented with 10% heat inactivated fetal calf serum (HyClone, USA), 2 mM of l-glutamine (PanEco, Russia) and antibiotic antimycotic solution (Millipore-Sigma, USA) in a humidified atmosphere of 5% CO_2_ at 37°C.

### Immunostaining and Flow Cytometry

Cells were stained with a combination of monoclonal antibodies (mAbs) and analyzed as described earlier (39). Intracellular IFN-γ staining was performed using the Inside Stain Kit (Miltenyi Biotec, Germany).

The following mAbs were used for staining: CD3-PE-Cy7 (clone UCHT1), CD56-APC (clone N901), CD56-PE (clone N901), CD45RO-PE (clone UCHL1) from Beckman Coulter; CD56-Brilliant Violet 421 (clone HCD56), CD56-PE-Cy7 (clone 5.1H11), anti-HLA-DR-PE (clone L243) from Sony Biotechnology; NKG2C-AlexaFluor (AF) 488 (clone 134591), NKG2C-PE (clone 134591), NKG2A-PE (clone 131411) from R&D Systems; CD45RA-PE (clone HI100), γδTCR-BrilliantViolet 421 (clone B1), γδTCR-PE (clone B1), CD161-AF647 (clone HP-3G10), CCR7-PE (clone G043H7) from Biolegend; CD45RA- Brilliant Violet 421 (clone HI100), CD57-PE (clone TB01), CD69-PE (clone FN50), anti-NKG2D-PE (clone 1D11) from eBioscience; CD16-FITC (clone REA423), CD45RO-FITC (clone UCHL1), CD57-APC (clone TB03), KIR2DL2/DL3-PE (clone DX27) from Miltenyi Biotech; CD3-APC (BD Biosciences, USA, clone UCHT1), CD4-FITC (Sorbent, RF, clone 1), CD8-PerCP Cy5.5 (Immunotech, France, clone B9.11), CD27-PE (Invitrogen, USA, clone CLB-27/1); anti-mouse IgG F(ab′)_2_-PE (Sigma, USA). Supernatant of CD28-specific hybridoma was kindly provided by Prof. Miguel Lopez-Botet (Universitat Pompeu Fabra, Barcelona, Spain).

Multicolor flow cytometric analysis was performed on a FACSCalibur flow cytometer equipped with 488 and 640 nm lasers and a FACSVantage DiVa cell sorter equipped with 405, 488, and 643 nm lasers (BD Biosciences, USA). Lymphocytes were gated according to forward and side scatter. 0.5 to 1 × 10^6^ events were recorded in this gate for PBMC phenotypic analysis. Data were analyzed using FlowJo version 7.6 and 10 (FlowJo LLC, USA).

### Fluorescence-Activated Cell Sorting

In most experiments, freshly isolated PBMC stained with anti-CD3, anti-CD56, and anti-NKG2C antibodies were subjected to fluorescence-activated cell sorting using a FACSVantage DiVa cell sorter. CD3^+^CD56^−^NKG2C^−^, CD3^+^CD56^−^NKG2C^+^, CD3^+^CD56^+^NKG2C^−^, CD3^+^CD56^+^NKG2C^+^ cells were separated into 12 × 75 mm tubes containing serum-free medium. In several experiments anti-γδTCR mAb was used to isolate only αβTCR-expressing T cells. Cell sorting was carried out at a sheath pressure of 27 psi using a 70-μm nozzle. Cell purity following cell sorting always exceeded 95%. For RNA isolation, sorted subpopulations were transferred into Trizol reagent (Thermo Fisher Invitrogen, USA) and frozen. Another portion of cells was transferred into cultivation medium AIM-V (Gibco-Thermo Fisher Scientific, USA) and then used in functional experiments.

### TCR cDNA Library Preparation, Sequencing, and Data Analysis

Libraries of TCR beta chains were prepared as previously (40). In brief, total RNA was isolated from PBMC using Trizol reagent. Isolated RNA was used for cDNA synthesis with 5′RACE template switch technology to introduce universal primer binding site and Unique Molecular Identifiers (UMI) at the 5′ end of RNA molecules. Primers complementary to the TCR-beta constant segments were used for cDNA synthesis initiation. cDNA was amplified in two subsequent PCR steps. The resulting libraries were sequenced on the Illumina HiSeq 2000/2500, MiSeq or NextSeq platform with 2 × 150 bp sequencing length. Raw sequencing data are deposited in the ArrayExpress database (E-MTAB-9601).

Sequencing reads were demultiplexed and clustered by UMI with MIGEC software (41). MiTCR software was used to assemble the TCR clonotype sequences, count clonotype proportion and form the final list of identified clonotypes for each sample (42). To exclude most erroneous TCR sequences, only those TCR cDNA sequences which were covered by at least two reads were used. Further TCR repertoire analysis was carried out using tcR package (43). The clonotypes belonging to different subsets of T cells were determined by the coincidence of the nucleotide sequence of the CDR3 region and the V-segment of TCR between the repertoires of the T cell fractions. TCR annotation to known antigen-specificity was performed using the VDJdb database (44) with a single substitution allowed in the CDR3 amino acid sequence using VDJmatch software (https://github.com/antigenomics/vdjmatch).

### Cytotoxicity Tests

To estimate functional NK-like responsiveness, the CD56^+^/^−^ NKG2C^+^/^−^ T cell subsets isolated by sorting were pre-incubated for 18 h in AIM-V medium supplemented with 10% fetal calf serum and 500 U/mL of recombinant IL-2 at a concentration of 2 × 10^5^ cells/ml. At the end of incubation, the cells were harvested and subjected to functional assays.

### Natural Cytotoxicity Assay

Cytolytic potential was assessed by degranulation assay based on LAMP-1 (CD107a) cell surface mobilization following T and NKT cells incubation with MHC class I-negative K562 target cells. Effectors were mixed with K562 cells in 1:1 ratio, centrifuged shortly and incubated for 3 h in the presence of PE-Cy5-conjugated anti-human CD107a antibody (eBioscience, USA, clone H4A3) and brefeldin A (10 μg/ml, Sigma, USA). Samples without target cells were included as a control of spontaneous degranulation. Then cells were washed with staining buffer and analyzed by flow cytometry.

### Antibody-Dependent Cell-mediated Cytotoxicity Assay

IL-2–activated T cells were mixed with C1R target cells in 3:1 ratio and incubated in RPMI-1640 medium with anti-CD20 mAb Rituximab (Roche Holding, Switzerland) in the presence of 10 μg/ml of brefeldin A and CD107a mAb for 2.5 h. Samples without target cells or without Rituximab were used as negative controls. After the incubation, cells were harvested and analyzed by flow cytometry.

### Statistical Analysis

Mann-Whitney U-test was used for determination of statistical significance of the differences. P-values of < 0.05 were considered significant.

## Results

### Expression of NKG2C in T Cells Is Associated With the Expression of NKG2C in NK Cells and the Development of a Pool of CD56^+^ T Cells

**Figure 1:**
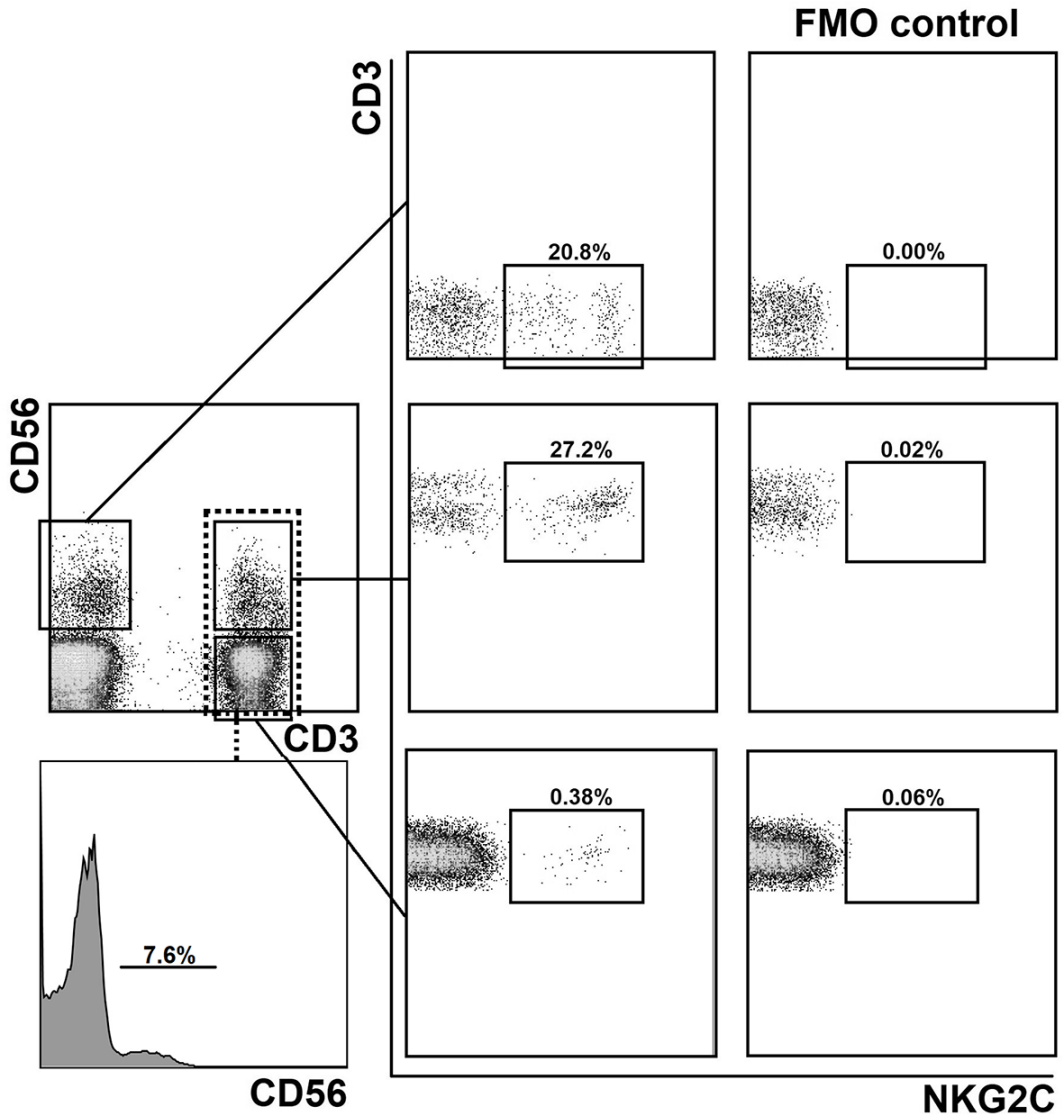
Gating strategy for the assessment of the distribution of NKG2C^+^ cells in CD56^−^ and CD56^+^ (NKT-like) T cell subsets and in NK cells. Experimental sample and fluorescence minus one (FMO) control lacking NKG2C-antibody are presented.

First, we examined the distribution of CD56^+^ and NKG2C^+^ T cell subsets in PBMCs of healthy donors (20–60 years old). 35 samples were collected and analyzed (24 CMV^+^, age median 23, and 11 CMV^−^, age median 27). PBMC were stained for CD3, CD56 and NKG2C antibodies, and analyzed by flow cytometry. The gating strategy is presented in **Figure 1**. The proportion of NKT-like cells (CD56^+^ T cells) among CD3^+^ cells ranged from 2 to 25% (mean 7.7%). The proportion of NKG2C^+^ cells among T lymphocytes averaged 2.0%. At the same time, among CD56^+^ T cells, NKG2C^+^ cells averaged 11.3%, but this value varied greatly (from 0.4% to 40.4%). In CD56-seronegative T cell subset, the proportion of NKG2C^+^ cells was less than 1%. In all analyzed T cell subsets the frequency of NKG2C+ cells was higher in HCMV-positive individuals compared to HCMV-seronegative donors. In more than 50% of donors, NKG2C^+^ T cells predominated in the more differentiated CD56^+^ fraction in both relative and absolute numbers; thus, the ratio of CD3^+^CD56^+^NKG2C^+^ cells to CD3^+^CD56^−^NKG2C^+^ cells was greater than 1. Most of the donors in the study group were HCMV-seropositive, comprising approximately 69% of all analyzed subjects. At the same time, in all seronegative individuals the indicated ratio was less than 1.

A strong correlation was observed between the size of the CD56^+^ NKT-like cell fraction and the proportion of NKG2C^+^ cells among T cells in HCMV-seropositive individuals (**Table 1**). There was a closer association in this group between the proportion of CD56^+^ cells among all T cells and the proportion of NKG2C^+^ cells among CD56-negative T cells (r = 0.671, p = 0.0002) than the association between the proportion of CD56^+^ cells among all T cells and NKG2C^+^ cells among CD56^+^ T cells (r = 0.533, p = 0.0037). The proportion of NKT-like cells correlated modestly with the proportion of NKG2C^+^ cells in the NK cell population in the group of HCMV-seropositive donors. Interestingly, in the group of HCMV-seronegative donors a negative correlation between CD56^+^ T cell frequency and NKG2C^+^ cells in this subset was observed. A modest positive correlation of NKG2C expression in T cells with NKG2C expression in NK cells was identified in HCMV-seropositive individuals (r = 0.411, p = 0.023). Interestingly, for HCMV-seronegative individuals, a strong negative correlation was identified between the NKG2C+ NK cell frequency and the NKG2C^+^ cell frequency in CD56-negative T cell subset.

**Table 1:** The distribution of NKT-like cells among T cells and of NKG2C^+^ cells in CD56^−^, CD56^+^ T cell subsets and NK cells in HCMV-positive and negative individuals, and the respective correlation analysis.

			CD3^+^: CD56^+^ (%)	CD3^+^: NKG2C^+^ (%)	CD3^+^CD56^−^: NKG2C^+^ (%)	CD3^+^CD56^+^: NKG2C^+^ (%)
Mean ± SD		CMV+/−	7.73 ± 7.38	1,99 ± 3,80	0.74 ± 1.04	11.25 ± 13.99
		CMV+	9.21 ± 8.27	2,71 ± 4,42	0.89 ± 1.22	14.73 ± 15.68
		CMV−	4.5± ±,36	0,43 ± 0,27	0.4 ± 0.26	3.67 ± 2.82
Correlations						
CD3^+^: NKG2C^+^ (%)		CMV+/−	r = 0.705 p = 2E-07			
		CMV+	r = 0.785 p = 2Е-07			
		CMV−	Ns			
CD3^+^CD56^−^: NKG2C^+^ (%)		CMV+/−	r = 0.496 p = 0.0012			
		CMV+	r = 0.671 p = 0.0002			
		CMV−	ns			
CD3^+^CD56^+^: NKG2C^+^ (%)		CMV+/−	r = 0.382 p = 0.012			
		CMV+	r = 0.533 p = 0.0037			
		CMV−	r = −0.6 p=0.028			
CD3^−^CD56^+^: NKG2C^+^ (%)		CMV+/−	r = 0.421 p = 0.006	r = 0.441 p = 0.004	ns	r = 0.447 p = 0.0036
		CMV+	r = 0.411 p = 0.023	r = 0.411 p = 0.023	ns	r = 0.347 p = 0.048
		CMV−	ns	ns	r = −0.7 p = 0.01	ns

Thus, the expression of NKG2C in CD56-negative T cells was strongly associated with the development of a pool of NKT-like cells. Moreover, the expression of NKG2C was coordinated in the T cell and NK cell compartments under HCMV infection to a certain extent, but could be oppositely associated in HCMV-seronegative individuals.

### The Distribution of TCR Beta-Chains in PBMC Fractions Is Associated with Clonal Cell Expansion

**Figure 2:**
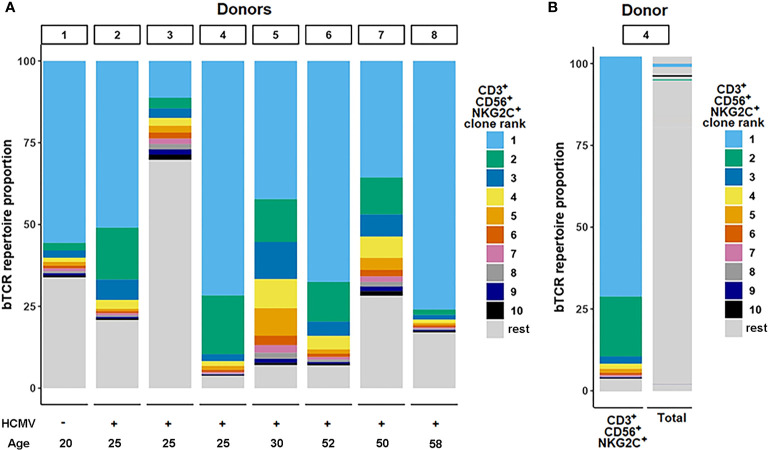
TCR-beta repertoire structure. **(A)** Repertoire structure of the CD3^+^CD56^+^NKG2C^+^ fraction. Ranking of clonotypes was performed in the cell fraction isolated by fluorescence-activated cell sorting. Top 10 clonotypes identified in samples for donors are shown proportionally of their percentage in the fraction. **(B)** The top clonotypes of the CD3^+^CD56^+^NKG2C^+^ fraction matched in total TCR-beta repertoire sequenced from PBMC sample.

We analyzed clonal repertoires of the CD3^+^CD56^+^ T cell subsets, negative or positive for NKG2C, and CD3^+^CD56^−^NKG2C^+^ subset in eight randomly selected donors (three men, five women) using HTS profiling of TCR beta-chain repertoire (**Supplementary Tables 1**, **2**). Seven donors were HCMV-seropositive, and one was HCMV-seronegative. In all eight donors one or several expanded TCR clonotypes represented most T cells of the CD3^+^CD56^+^NKG2C^+^ cell fraction (proportion of the most expanded clonotype was 11.3% to 76%, mean 51.3%, n = 8) (**Figure 2A**). Interestingly, in the HCMV-seronegative individual the most expanded clonotype represented more than 50% T cells in this fraction. The CDR3β nucleotide sequence of the dominant clone was unique for each donor (**Supplementary Table 2**), with specific V-segments and relatively long NDN regions of CDR3 for most of the donors. We have not found a significant similarity of the clonotypes with the known MAIT cell TCR beta chains according to the MAIT Match Server database (45) or, for most of clonotypes, with the matching TCRs in VDJdb database, which contains TCR sequences with characterized antigen specificity (44). Importantly, in four donors the same clonotypes were also the most frequent in the CD3^+^CD56^−^NKG2C^+^ fraction (28.3–60.1%) (**Supplementary Tables 1**, **2**), which may be considered as a fraction of less differentiated cells compared to the CD3^+^CD56^+^NKG2C^+^ subset. Then we analyzed the distribution of the identified clonotypes in clonal repertoires of CD4^+^/CD8^+^ T cells. For six donors the clonotypes identified as dominant in CD3^+^CD56^+^NKG2C^+^ subset were found within most expanded (top 10) CD8^+^ T cell clonotypes, and represented significant proportion (1.0–7.0%) of CD8^+^ T cells (**Supplementary Tables 1**, **2**). In donor 2, the clonotype prevailing in the CD3^+^CD56^+^NKG2C^+^ subset (50.9%) was found to be dominant in the repertoire of CD4^+^ T cells (15.9%). For six donors, clonal repertoires of the bulk peripheral blood T lymphocytes were also studied (**Supplementary Table 1**). For the analyzed donors, the TCR clonotypes prevailing in the CD3^+^CD56^+^NKG2C^+^ fraction were also identified between most expanded clonotypes of peripheral blood T cells (**Figure 2B**; **Supplementary Table 2**). Notably, the same clonotypes were also found in the repertoire of CD56^+^NKG2C^−^ T cells with various frequencies, indicating that the T cell clones were not 100% NKG2C-positive and may acquire NKG2C at certain stages of the cell differentiation (**Supplementary Table 2**).

To summarize, we found that the CD3^+^CD56^+^NKG2C^+^ subset had an oligoclonal TCR repertoire in all donors studied. Moreover, the dominant clonotypes of this subset represented a significant proportion of the whole peripheral blood αβ T cell population. In most cases the clonotypes were attributed to the CD8^+^ T cells. The data suggest that these clones underwent expansion during which they partly acquired surface NKG2C. Apparently, this expansion could be attributed to the inflation previously described for CMV-infected individuals (26).

### NKG2C^+^ T Cells Display the Phenotype of Highly Differentiated Cells

**Figure 3:**
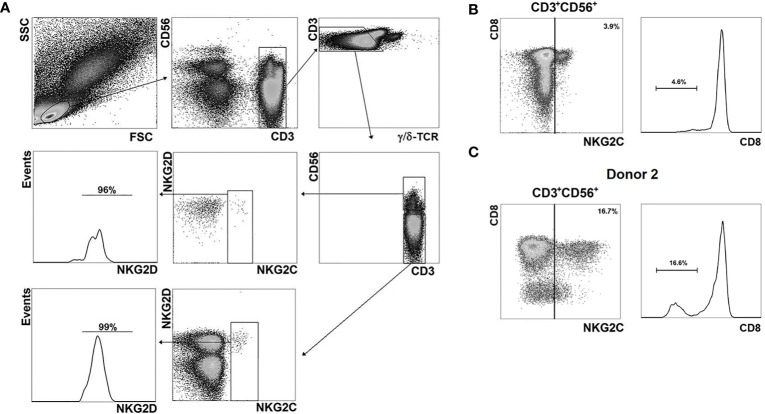
Phenotypic analysis of NKG2C^+^ cells. **(A)** Gating strategy for the assessment of the distribution of various surface markers in NKG2C^+^ cells in CD56^−^ and CD56^+^ αβ T cell subsets. As an example, NKG2D expression level in the T cell fractions is presented; antibody panel: CD3-PE-Cy7, CD56-APC, γδTCR-Brilliant Violet 421, NKG2C-AF488, NKG2D-PE. **(B)** Analysis of CD8 expression in CD56^+^NKG2C^+^ αβ T cells. Representative staining is presented. **(C)** Analysis of CD8 expression in CD56^+^NKG2C^+^ T cells of the donor 2.

**Figure 4:**
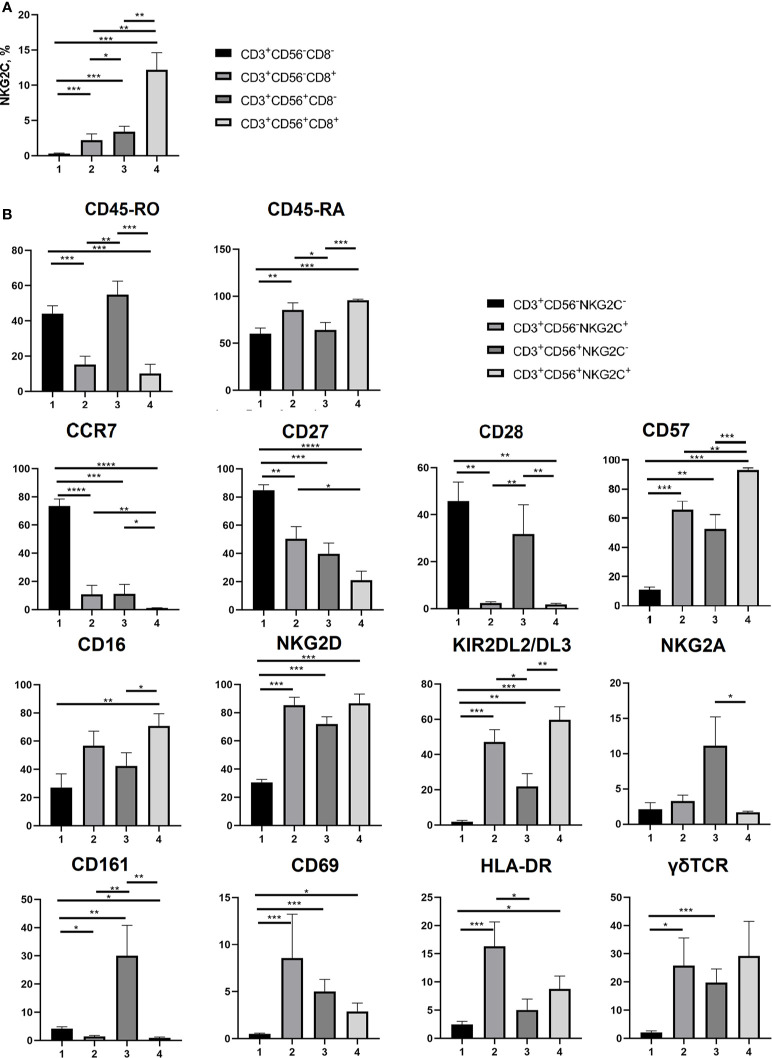
Phenotypic analysis of T cell subsets. **(A)** NKG2C+ cell frequencies analyzed in CD3+CD56+/−CD8+/− cell subsets (n = 7). **(B)** Expression of CD45-RA, CD45-RO, CCR7 (CD197), CD27, CD28, CD57, CD16, NKG2D, KIR2DL2/DL3, NKG2A, CD161, CD69, HLA-DR and γδTCR on NKG2C^−^ and NKG2C^+^ CD56^−^/^+^ T cells (n = 8, 9, 9, 9, 5, 7, 8, 7, 8, 7, 5, 8, 8 and 8, respectively. **(B)** NKG2C+ in CD8^+/−^CD56^+/−^ T cell subsets (n = 7). Means ± SEM are presented (*p < 0.05; **p < 0.01; ***p < 0.001; ****p < 0.0001, Mann-Whitney U test).

We characterized the phenotype of NKG2C^+^ cells among CD56^−^ and CD56^+^ T cells by multicolor flow cytometry (**Figure 3**). In all PBMC samples the majority of CD56^+^NKG2C^+^ αβ T cells were CD8^+^ (**Figure 3B**; **Figure 4A**). Notably, donor 2 had more than 15% CD8^−^NKG2C^dim^ T cells of the CD56^+^NKG2C^+^ fraction (**Figure 3C**).

Comparative analysis of a number of surface markers was performed in CD56^−^NKG2C^−^, CD56^−^NKG2C^+^, CD56^+^NKG2C^−^, and CD56^+^NKG2C^+^ subsets of CD3^+^ cells. It included the measurement of expression level of the T cell differentiation markers CD45RA, CD45RO, CCR7, CD27, and CD28, the marker of terminal differentiation (or exhaustion) of NK and T cells CD57, NK cell receptors NKG2D, CD16, KIR2DL2/DL3, NKG2A, CD161, and NK and T cell activation markers CD69 and HLA-DR (**Figure 4B**; **Supplementary Figure 1**). The expression of γδTCR was also tested in these fractions (**Figure 4B**).

Our data supported the observation that CD56^+^ T cells were more differentiated compared to the CD56^−^ T cells. In particular, they were characterized by almost complete lack of surface CCR7, higher expression level of CD57, and lower level of CD27 (**Figure 4**; **Supplementary Figure 1**). Strict differences were observed between NKG2C^−^ and NKG2C^+^ cells within both CD56^+^ and CD56^−^ T cell fractions. NKG2C^+^ subsets contained significantly larger proportions of cells expressing CD45RA and CD57, and smaller proportions of CD45RO^+^, CD27^+^, and CD28^+^ cells. Remarkably, even in the fraction of CD56^−^ T cells, most of which are CCR7^+^, the NKG2C^+^ cells were almost completely negative for CCR7 expression (**Figure 4**; **Supplementary Figure 1**). Thus, most of the NKG2C^+^ cells demonstrated the phenotype of terminally differentiated TEMRA cells (CCR7^−^CD45RA^+^CD27^−^CD28^−^) and expressed CD57 surface marker related to the terminal cell differentiation and low proliferative activity. CD56^+^NKG2C^+^ cells were negative for CD161 expression, whereas a significant proportion of CD56^+^NKG2C^−^ cells expressed this receptor. CD161 expression was shown earlier to be associated with mucosal invariant T cells (MAIT) (46).

NKG2C^+^ T cells in both CD56^−^ and CD56^+^ subsets demonstrated significant levels of activating NK cell receptors NKG2D and CD16 and inhibitory receptors KIR2DL2/DL3. High percentages of cells expressing the receptors mentioned above is typical for CD56^+^ T cells (13, 14), but NKG2C^+^ T cells in this subset had even higher expression levels of these receptors. At the same time, NKG2C-positive T cells in the CD56^+^ fraction had almost no expression of NKG2A, similarly to NKG2C^+^NKG2A^−^ adaptive-like NK cells (47).

CD56^−^NKG2C^+^ T cells had higher expression levels of CD69 and HLA-DR, thus showing more activated state than T cells from the most numerous CD56^−^NKG2C^−^ subset (**Figure 4**; **Supplementary Figure 1**). Notably, CD56^+^NKG2C^+^ T cell subsets of certain donors had no CD69^+^ or HLA-DR^+^ cells.

### Analysis of CMV-Specific Response of NKG2C+ NK Cells

**Figure 5:**
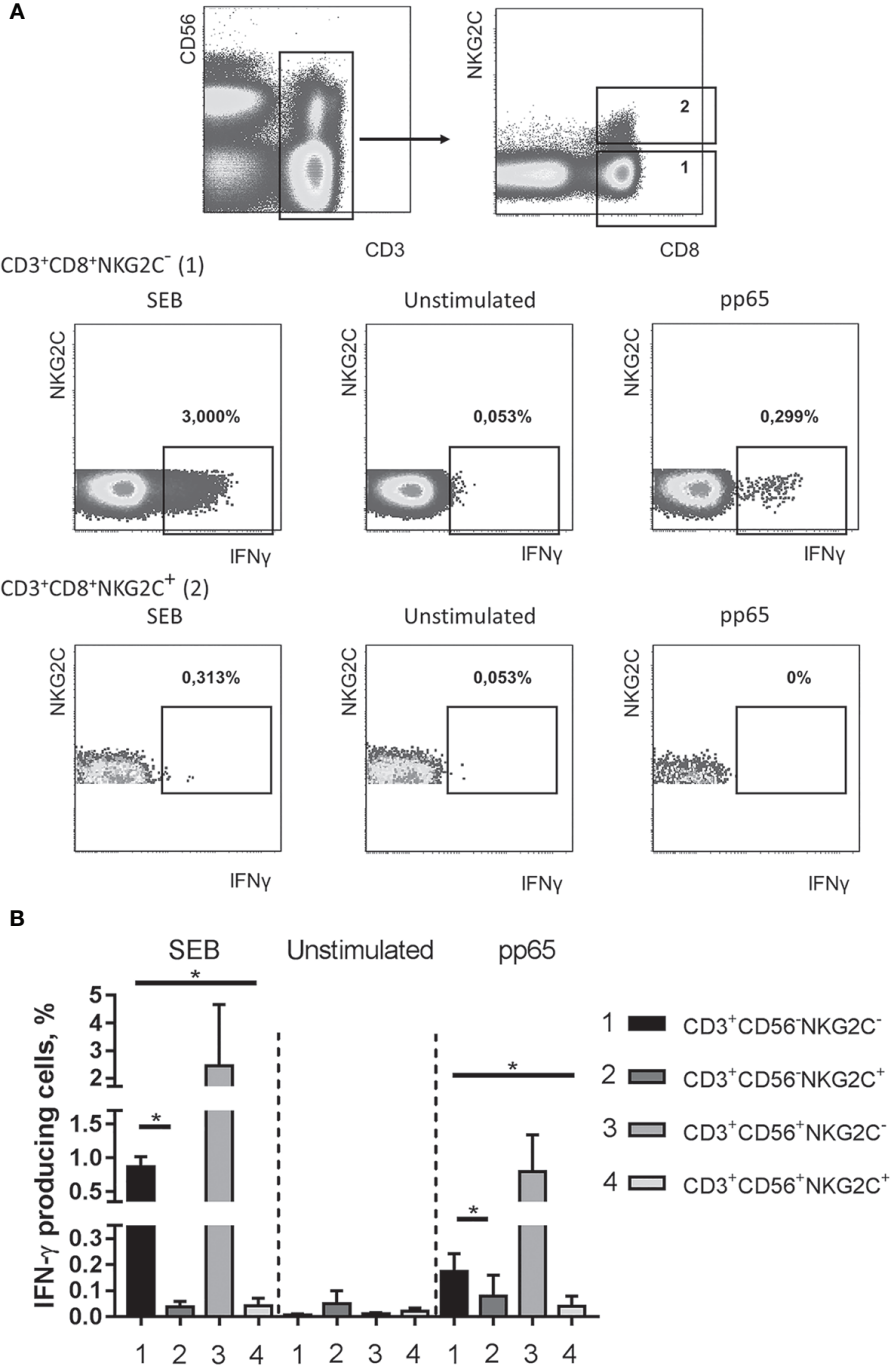
Production of IFN-γ in NKG2C^−^ and NKG2C^+^CD8^+^ T cells. **(A)** Representative staining of cells from one of the donors is presented. CMV-specific IFN-γ response was induced using pp65 peptide set (PepTivator, Miltenyi Biotec). Staphylococcal enterotoxin B was used as a positive control. **(B)** Summary of data on IFN-γ production accessed in CD56^−^NKG2C^−^, CD56^−^NKG2C^+^, CD56^+^NKG2C^−^, CD56^+^NKG2C^+^ cells from the CD3^+^CD8^+^ T cell subset. Means ± SEM of three independent experiments are presented. *p < 0.05.

We then evaluated IFN-γ production in NKG2C-negative and NKG2C-positive CD8^+^CD56^+^/^−^ T cells in response to stimulation with a set of peptides from HCMV pp65 protein (PepTivator, Miltenyi Biotec). CD3^+^CD8^+^NKG2C^−^, but not CD3^+^CD8^+^NKG2C^+^ cells showed IFN-γ response to both CMV peptides and staphylococcal enterotoxin B (SEB) (as an unspecific control), although production levels varied among donors (**Figure 5A**). Interestingly, a significant number of IFN-γ-producing cells could be found in a subset of CD56^+^NKG2C^−^ T cells, thus indicating the capability of cells at this differentiation stage for antigen-specific response (**Figure 5B**). There were almost no IFN-γ producers in the CD8^+^CD56^+^NKG2C^+^ subset that demonstrated the lack of pp65-specific response in this cell fraction. On the other hand, when staphylococcal enterotoxin B (SEB) was used for the stimulation, the IFN-γ response level of the NKG2C^+^ T cells was lower compared to NKG2C^−^ T cell response (**Figure 5**). SEB is known to unspecifically bind to a number of TCRβ chain variants (48–50). While NKG2C^+^ T cells were shown to be oligoclonal, their lack of response to SEB might reflect their inability to bind the superantigen. However, the TCRβ chain repertoire analysis revealed that at least one variant of TCRβ chain that should bind SEB was found within the top clonotypes of the CD56^+^NKG2C^+^ fraction in seven of eight analyzed individuals (**Supplementary Table 3**).

**Figure 6:**
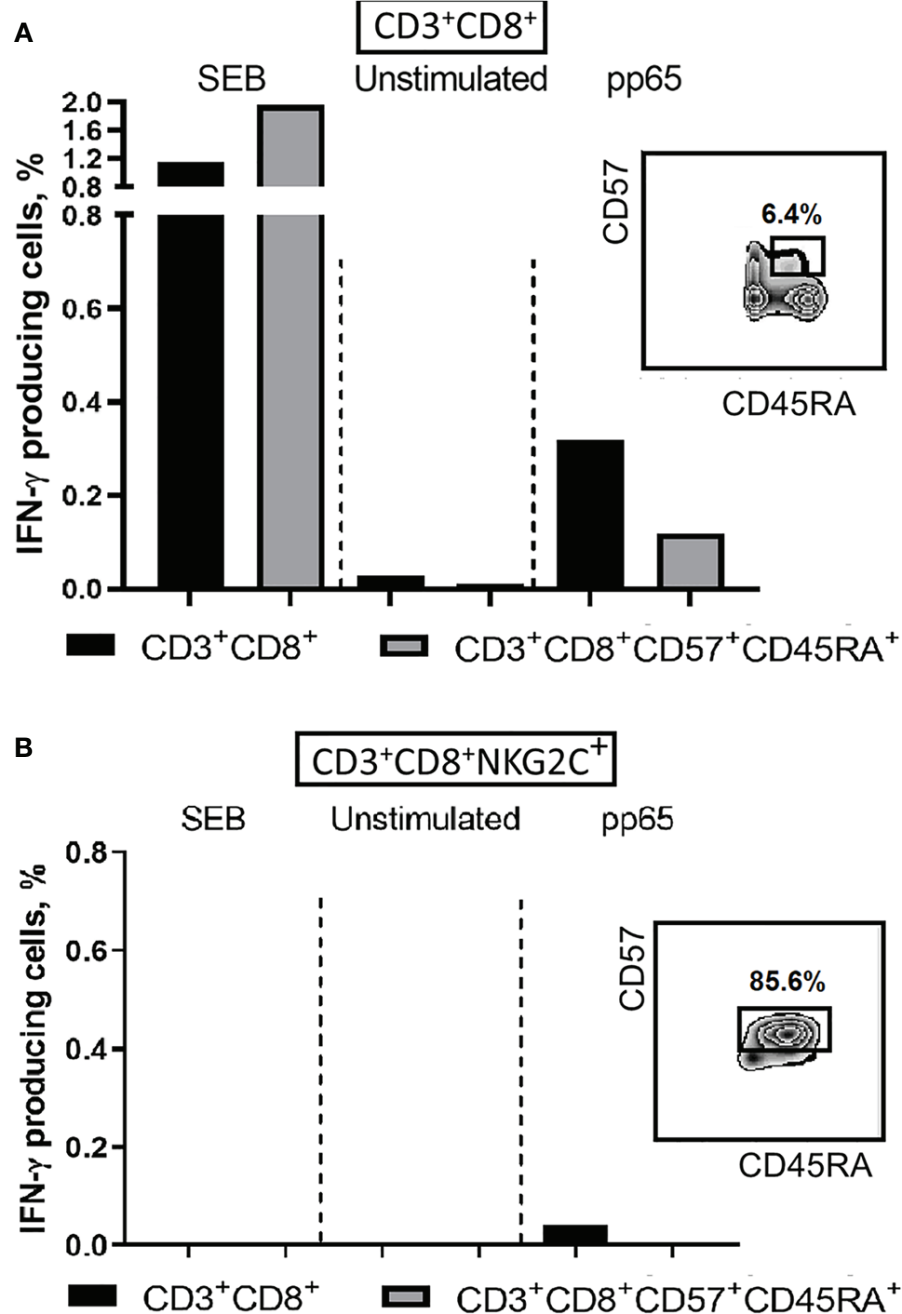
IFN-γ production induced by SEB (positive control) or pp65 peptide set in CD8^+^ T cells. **(A)** IFN-γ production levels in CD8^+^ and more differentiated CD8^+^CD57^+^CD45RA^+^ T cell subsets. **(B)** IFN-γ production levels in CD8^+^NKG2C^+^ T cell subsets and in more differentiated CD8^+^CD57^+^CD45RA^+^NKG2C^+^ T cells. Percentages of CD57^+^CD45RA^+^ cells in CD8^+^ and CD8^+^NKG2C^+^ cell fractions are indicated. Representative staining is presented.

To address either the decrease in the antigen-specific response in T cells is related to the acquisition of NKG2C expression or to the terminal differentiation, we analyzed the production of IFN-γ in NKG2C^+^/^−^ CD8^+^ T cells co-expressing CD57 and CD45RA. Among the CD8^+^ T cells various ratios of cells expressing CD45RA and CD57 were found in different donors, and the CD45RA^+^CD57^+^ cells produced less IFN-γ in response to pp65, compared to all CD8^+^ T cells (**Figure 6A**). On the other hand, while CD45RA^+^CD57^+^ cells seemed to retain a certain level of antigen-specific IFN-γ production, the NKG2C^+^ cells, most of which were positive for CD45RA and CD57, produced almost no IFN-γ (**Figure 6B**).

The obtained data indicate that T cell differentiation was associated with decline in the antigen-specific IFN-γ production with the minimal production in NKG2C-expressing cells. The expression of NKG2C indicated the expansion and “aging” of individual T cell clones. However, it remains unclear whether such clones were specific for HCMV antigens.

### NK Cell-Like Effector Activity of NKG2C-Expressing T Cells

**Figure 7:**
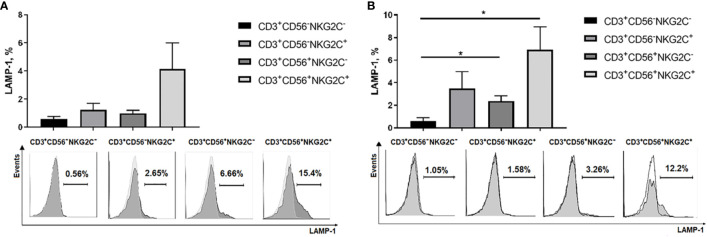
Cytotoxic activity of NKG2C^−^/^+^ CD56^−^/^+^ T cells estimated by surface expression of LAMP-1 in degranulation assay. **(A)** Natural cytotoxicity of T cells measured against K562 target cells. T cell subsets isolated from PBMC by cell sorting were stimulated overnight with IL-2 (500 U/ml). Percentage of LAMP-1–positive T cells after incubation with target cells was measured. Means ± SEM of 6 independent experiments and histograms of one of the experiments are presented. **(B)** Antibody-dependent cell-mediated cytotoxicity of T cells measured against Rituximab-coated C1R target cells. Means ± SEM for 5 independent experiments and histograms of one of the experiments are presented. *p < 0.05.

Natural cytotoxic activity was estimated in CD3^+^CD56^−^NKG2C^−^, CD3^+^CD56^−^NKG2C^+^, CD3^+^CD56^+^NKG2C^−^, and CD3^+^CD56^+^NKG2C^+^ subsets in degranulation assay using K562 cell line, the standard cytotoxicity target for human NK cells. No degranulation was observed in the CD3^+^CD56^−^NKG2C^−^ cell subset. The remaining three subsets showed weak natural cytotoxic activity; the highest natural cytotoxicity level was observed among NKG2C-expressing more differentiated CD56^+^ T cells (**Figure 7A**). The CD56^+^NKG2C^+^ T cell subset was also characterized by a higher level of antibody-dependent cell-mediated cytotoxicity (ADCC) measured against Rituximab-coated C1R cells (**Figure 7B**).

## Discussion

The whole population of peripheral T cell consists of many subsets, combinations of which seem to meet different requirements for an effective immune response against a variety of infections. In this work, we characterized T cells which express molecules characteristic of NK cells, primarily the CD56 molecule (a distinctive marker of NK cells that functions as an adhesion receptor) (51). In our cohort of donors, the average number of CD56-expressing, or NKT-like, cells was 8.5% of all CD3^+^ cells. CD56, as well as a number of other NK cell receptors (including receptors of the KIR family, NKG2D, and CD16), can be expressed on T cells at the late stages of differentiation, when they lose CD28 expression. It was also shown that stimulation of CD8^+^ T cells from a healthy donor with melanoma antigenic peptides (tyrosinase and mart-1) *in vitro* produces T cell lines expressing KIR2D and polyclonal TCR (4). Therefore, it could be assumed that the expression of NK cell receptors appeared at a certain stage of T cell differentiation during the formation of an antigen-specific response.

Highly differentiated CD56^+^ T cells are rather heterogeneous. One of the subsets of these cells is determined by the presence of the activating NKG2C receptor, the increased expression of which in NK cells is associated with HCMV infection. CD56^+^NKG2C^+^ subset can be found among peripheral blood T cells of healthy donors (28). In our study, the size of this subset could reach 25% in certain individuals (**Table 1**). Most of these cells expressed CD57 and CD45RA in the absence of CD45RO and CCR7, confirming that they belonged to the pool of highly differentiated TEMRA cells. A significant part of CD56^+^ T cells had high expression of CD161, characteristic of MAIT cells (46), although negligible numbers of CD161^+^ cells were detected in the CD56^+^NKG2C^+^ fraction.

Since CD56^+^ T cells have previously shown a reduced ability to divide due to increased levels of proteins that regulate cell cycle (Bcl2, p16, p53) and transcription factors that control mitogen-mediated activation (znf394, gdpd5, tfcp2l1, and snhg12), and decreased levels of transcription factors involved in DNA replication (tyms, rpa1, tmf1, and ecop) (12, 13), the accumulation of such cells in the peripheral blood should be associated with their better survival. Indeed, increased resistance to apoptosis of CD56^+^ T cells has been shown previously (12, 13). Although accumulation of T cells expressing CD56 is observed during certain pathologies (e.g., malaria) (52), an increase in this subset can also occur with age, especially with concomitant HCMV infection (12).

The accumulation of CD8^+^CD56^+^ T cells and NKG2C-positive NK cells in HCMV-infected individuals is of great interest (28, 53). The size of these subsets in the peripheral blood can increase with age even in HCMV-seronegative individuals (8). Early data supported an association of HCMV effects on NKG2C^+^ T and NK cell subsets (28). Another work showed that these responses are interrelated (8). In particular, correlations were found: 1) between the frequency of CD57^+^ T cells and CD57^+^ NK cells (r = 0.5) 2) between the frequency of IFN-γ-producing T cells in response to stimulation with peptides (pp65, IE1) and the frequency of NKG2C^+^CD57^+^ NK cells (r = 0.34). Several other studies reported largely independent and overlapping effects of HCMV on CD8 T cells and NK cells (54, 55). In another study, there appeared to be no direct relationship between the T cell and NK cell response to HCMV, however, there was an inverse correlation between the frequency of virus-specific T cells and the frequency of NKG2C^+^CD57^+^ NK cells during HCMV and HIV co-infection (56). Recently, it has been suggested that HCMV infection triggers an NKG2C^+^ NK cell response, or a T cell response, but not both (55). In our work, a positive correlation has been revealed between the frequency of highly differentiated CD56^+^ T cells and the frequency of NKG2C^+^ T cells in HCMV-seropositive individuals, which implies that the development of these T cell subsets is partly interrelated. Moreover, in this group of donors we found a relationship between the content of NKG2C-expressing cells in the T and NK cell fractions, although the correlation coefficients were less than 0.5 (**Table 1**). It suggests that there should be common conditions and mechanisms for their formation. It is known that adaptive NK cells acquire stable epigenetic changes in gene regulation during their development and downregulate the expression of intracellular signaling molecules SYK and EAT-2, characteristic of myeloid cells, while the expression of ZAP-70 and SAP, typical of T cells, remains unchanged (57). At the same time, there is often a similarity in the pattern of gene methylation in adaptive NK cells and differentiated T cells (58). This similarity suggests a common transcriptional program, which is launched during the differentiation of CD8^+^ memory T cells and adaptive NK cells in response to HCMV. The varying amount of the NKG2C receptor ligand, HLA-E, in the organism possibly determines the relationship between the fractions of NKG2C^+^ T cells and NK cells. It can be assumed that continued contact with the antigen in the context of HLA-E under certain conditions promotes the expression of NKG2C and KIRs on the T cells (59). A negative correlation found in this work between NKG2C^+^ expression levels in NK cells and CD56-negative T cells in HCMV-seronegative individuals may be partly accounted for a possible explanation of the discrepancy of data on NKG2C^+^ T and NK cell subset association in different works.

We have revealed that a significant part (often the majority) of CD56^+^NKG2C^+^ T cells are the offspring of a single T cell clone. These data confirm the information obtained earlier and by other methods about the oligoclonality of CD8^+^ T cells that express MHC-I-interacting inhibitory receptors, characteristic of NK cells (60, 61). Interestingly, in one individual (donor 2) the clone, most abundant in the CD3^+^CD56^+^NKG2C^+^ fraction, was determined to be CD4^+^ based on the results of sequencing analysis. The sample contained a portion of CD8^−^ T cells with low expression of NKG2C, along with highly NKG2C-positive CD8^+^ T cells (**Figure 3C**). Apparently, at a certain stage of differentiation, a significant part of the cells of the clone acquired the expression of both CD56 and NKG2C, which allowed this clone to become the most represented in this fraction. The identified clones seem to have a significant lifespan: in one of the donors, the clone, most represented in the CD3^+^CD56^+^NKG2C^+^ fraction, has been registered for two years (62), but its frequency *in vivo* varied. The accumulation of such expanded clones seems to underlie the so-called “memory T cell inflation” described for HCMV-infected individuals (26, 27). It can be assumed that either a certain stimulus “warms up” the expansion of a certain clone for a long time, or a significant number of cells in a clone is due to their increased viability and, thus, accumulation in the peripheral blood.

Labeling of CD56^+^ T cells with antibodies to the NKG2C receptor allowed us in many cases to identify the clone that has undergone a great expansion in the αβ T lymphocyte population. We have shown that not only CD56-expressing, but also CD56-negative NKG2C^+^ T cells much more often than NKG2C^−^ cells have a phenotypic signature characteristic of TEMRA cells: high level of CD45RA, reduced level of CD45RO and CD27, and the absence of CCR7 and CD28 expression. Thus, NKG2C expression turned out to be typical for highly differentiated T cells. Earlier, Arlettaz et al. reported about NKG2C-positive T cells which belonged to effector cells with the CD45RA^+^CCR7^−^ phenotype (63). We have demonstrated that about a half of the NKG2C^+^CD56^−^ T cells expressed CD57, CD16 and KIR2DL2/3 on their surface, and more than 80% of these cells expressed the NKG2D receptor. All these phenotypic characteristics associated with NKG2C expression illustrate the formation of a pool of specialized NK-like effectors during T cell inflation. Interestingly, the largest percentage of both CD69^+^ and HLA-DR^+^ T cells was observed in the NKG2C^+^CD56^−^ fraction. Thus, it is this fraction, but not the most differentiated NKG2C^+^CD56^+^ fraction, which was activated in the process of T cell immune response. In a number of donors, CD69^+^ and HLA-DR^+^ T cells were almost absent; possibly, they were present in the peripheral blood at the earlier stages of the immune response.

Interestingly, NKG2C labeling allowed to identify one of the most significant clones of αβ T cells in an individual with undetectable level of anti-HCMV antibodies (according to two examinations separated in time by several months), although the size of the CD56^+^NKG2C^+^ fraction was slightly over 1%. It can be supposed that NKG2C may also mark clones of T (or NKT-like) cells that have undergone expansion induced during immune response not associated with HCMV infection. In this case, the level of expansion may be limited, and T cell inflation may not be observed. We should not exclude the possibility that this individual can be a carrier of HCMV infection even if the antibody response was not detected.

Overall, the phenotypic signature of CD56^+^NKG2C^+^ T cells, which for the most part acquired CD57 and KIR expression, but did not express NKG2A, was similar to the classic NKG2C^+^NKG2A^−^CD57^+^KIR^+^ adaptive NK cells (47). These data once again support the idea of convergence of the transcriptional programs for T cells and NK cells during differentiation in the presence of HCMV infection. It was previously demonstrated that T cells expressing NKG2A and NKG2C mostly belong to different subsets (63). We have revealed a significant number of NKG2A^+^ T cells in the CD56^+^NKG2C^−^ fraction. Expression of NKG2A seems to be required to control the autoreactivity of these cells. CD94 expressing T cells were discovered in the melanoma-specific T lymphocyte fraction (64). It has been shown recently that NKG2A-expressing T cells are partially responsible for the resistance of tumor cells to immune recognition (65, 66).

Although the literature contains many data on the functional activity of highly differentiated CD8^+^ T cells, these data are scattered and sometimes contradictory. Since we have revealed a certain coordination in the development of NKG2C^+^ T cells and adaptive NKG2C^+^ NK cell subsets, associated with cytomegalovirus infection, we made an attempt to evaluate the HCMV-specific response of highly differentiated CD8^+^NKG2C^+^ T cells using a set of peptides from one of the most immunogenic HCMV proteins pp65 (25). Also, we used staphylococcal enterotoxin as a common stimulus to induce T cell response, which is independent from the peptide loaded in the MHC I. Earlier CD56^+^ T cells were described to be effective IFN-γ producers contributing greatly to the antiviral responses (67). We demonstrated that CD56^+^ T cells do intensively produce IFN-γ in response to both pp65 peptides and SEB. However, both CD56^+^NKG2C^+^ and CD56^−^NKG2C^+^ T cell subsets produced IFN-γ to a much lesser extent than their NKG2C-negative counterparts. Thus, NKG2C^+^ T cell fractions showed no significant pp65-specific response in our system. Earlier, it was shown the NKG2C^+^ T cell expansion was not directly connected to conventional anti-HCMV response of T cells expressing pp65-specific TCRs (28). On the other hand, the low response of NKG2C^+^ T cells to both pp65 and SEB stimulation might be partly connected with a decreased ability of highly differentiated NKG2C^+^ T cells to produce IFN-γ. Thus, the intensity of IFN-γ production would depend on the phase of the immune response and decrease with the differentiation process. It is known that the functionality of the TCR can be disrupted in the highly differentiated T cells. Partially it may be a result of sustained antigen exposure leading to down-regulation of the T cell receptor zeta chain (68), which can explain lower IFN-γ production. According to another publication, no cells responding to HCMV by producing cytokines were found among KIR-positive T cells (61), which, according to our data, form about half of the NKG2C-expressing cells.

Nevertheless, it is still possible that the dominant clones we identified are HCMV-specific, since 1) these NKG2C-expressing cells were at the stage of differentiation (CCR7^−^CD45RA^+^CD57^+^), at which IFN-γ production is not the predominant type of antigen-specific response, and 2) these NKG2C-expressing cells could belong to αβTCR clones which are specific for another HCMV protein. Additionally, these cells still can be responsive to HLA-E stimulus, which is a ligand for the NKG2C receptor itself. The NKG2C molecule is expressed and can interact with HLA-E as a part of heterodimer with CD94 and in the presence of adapter DAP12 molecules. Co-expression of CD94 and DAP12 has been observed previously in NKG2C^+^ subsets of T cells, and interaction with HLA-E or specific antibodies caused proliferation and induction of CD25 expression in a small part of these cells (69). Finally, it is possible that the NKG2C^+^ T cell response is regulated by the presence of other receptors or requires co-stimulatory signals.

HLA-E-restricted T cells, characterized by monoclonality, have been previously observed too (70). It has been shown that HLA-E-restricted T cells accumulate upon HCMV infection, possess the CD45RA^+^CD28^−^CD27^−^CCR7^−^CD56^+^ phenotype and are able to recognize peptides derived from HCMV protein UL40 in the context of HLA-E (37, 71). The description of these cells is consistent with the characteristics of CD8^+^NKG2C^+^ T cells analyzed in this work. Recently, it was suggested that interaction with HLA-E by the HLA-E-restricted T cells happens partly *via* the αβTCR, since it was not blocked by anti-CD94 antibodies (72). Later, the same authors reported the molecular structures of TRBV9^+^TCR in complex with the HLA-E presenting the UL40 peptides (73). The pool of HLA-E-restricted T cells was found in about a third of HCMV-seropositive individuals and could account for up to 38% of circulating CD8^+^ T cells. It has been suggested that genetic factors in combination with the HCMV strain are essential for the emergence of this pool (38).

It is known that T cells contain increased amounts of perforin and granzymes at the late stages of differentiation, when CD28 expression is lost, which indicates their potentially high cytolytic activity (7, 11). A high correlation of cytolytic function with CD56 expression has been previously shown (11). CD56^+^ T cells which express NK cell receptors were able to lyse allogeneic NK-cell-susceptible targets (60, 70) and can demonstrate cytotoxic activity independently of the TCR (12). The data obtained in our study confirm a higher degranulation response of CD56^+^ T cells in the reactions of both natural and antibody-dependent cytotoxicity, compared with CD56-negative cells. Moreover, for NKG2C-positive T cells from both CD56^+^ and CD56^−^ fractions, there was a tendency toward a higher level of degranulation compared to NKG2C-negative cells. The level of cytotoxic response in the NKG2C^+^ T cell subsets from different individuals was quite variable, which may be explained by distinct stages of the immune response and, thus, differentiation state of these effectors at the moment of blood sampling.

Thus, we have shown that the clones of CD8^+^ αβ T lymphocytes, which have undergone preliminary expansion and are most represented in the peripheral blood, in most cases have a fraction of highly differentiated cells expressing the NKG2C receptor. These NKG2C^+^ cells are widely represented in the fraction of NK-like CD56^+^ T cells. Although the phenomenon of inflation is known for people infected with HCMV, we are not able to speak with confidence about the expansion of HCMV-specific T cells, since the recognition of virus-specific peptides in the context of classical HLA-I has not been presented. In addition, the intensity of the pp65-specific T cell response was earlier shown to correlate with the level of T cell differentiation in general, including non-HCMV-specific cells (74). In our experimental system, no pp65-specific functional response was detected in the NKG2C-expressing part of the dominant clone. Presumably, the expansion of T cell clones that do not recognize the standard viral peptides in the context of MHC I occurs due to interaction of certain TCRs with HLA-E in the presence of UL40-derived peptides, as it happens in the case of NK cells due to NKG2C-HLA-E interaction (75). Despite a number of publications indicating the existence of HLA-E-restricted T cells, the role of NKG2C receptor in the recognition of and interaction with HLA-E molecule bearing viral peptides remains intriguing. Perhaps a certain cooperation in the interaction of TCR and NKG2C with HLA-E leads to intensive proliferation of the clones co-expressing HLA-E-restricted TCR and NKG2C receptor. Possibly, the same stimulus, namely UL40 peptide exposed in the context of HCMV-induced HLA-E, underlies the proliferation, differentiation and accumulation of more differentiated NKG2C-expressing CD56^+^ TEMRA cells and adaptive-like NK cells.

## Data Availability Statement

The data sets presented in this study can be found in online repositories. The names of the repository/repositories and accession number(s) can be found below: https://www.ebi.ac.uk/arrayexpress/, E-MTAB-9601.

## Ethics Statement

The studies involving human participants were reviewed and approved by Pirogov Russian National Research Medical University. The patients/participants provided their written informed consent to participate in this study. 

## Author Contributions

Conception and study design – EK. Flow cytometry, cell sorting, correlation analysis, and functional studies — MS, AS, SE, and EK. Sequencing, reconstruction and analysis of TCR repertoires— IZ, AM, and YL. Manuscript editing AS, WT, and IZ. General expertise —YL. All authors contributed to the article and approved the submitted version.

## Funding

This work was supported by Russian Science Foundation. Research conception and design, flow cytometry, cell sorting, correlation analysis and functional studies were supported by grant #19-15-00439. Sequencing, reconstruction and analysis of TCR repertoires were supported by grant #20-15-00351.

## Conflict of Interest

The authors declare that the research was conducted in the absence of any commercial or financial relationships that could be construed as a potential conflict of interest.
