# Supplementary figures and images for: Surface NKG2C Identifies Differentiated αβT-Cell Clones Expanded in Peripheral Blood
Source: Front Immunol. 2021 Feb 16;11:613882. doi: 10.3389/fimmu.2020.613882 (PMC7921799; doi:10.3389/fimmu.2020.613882)

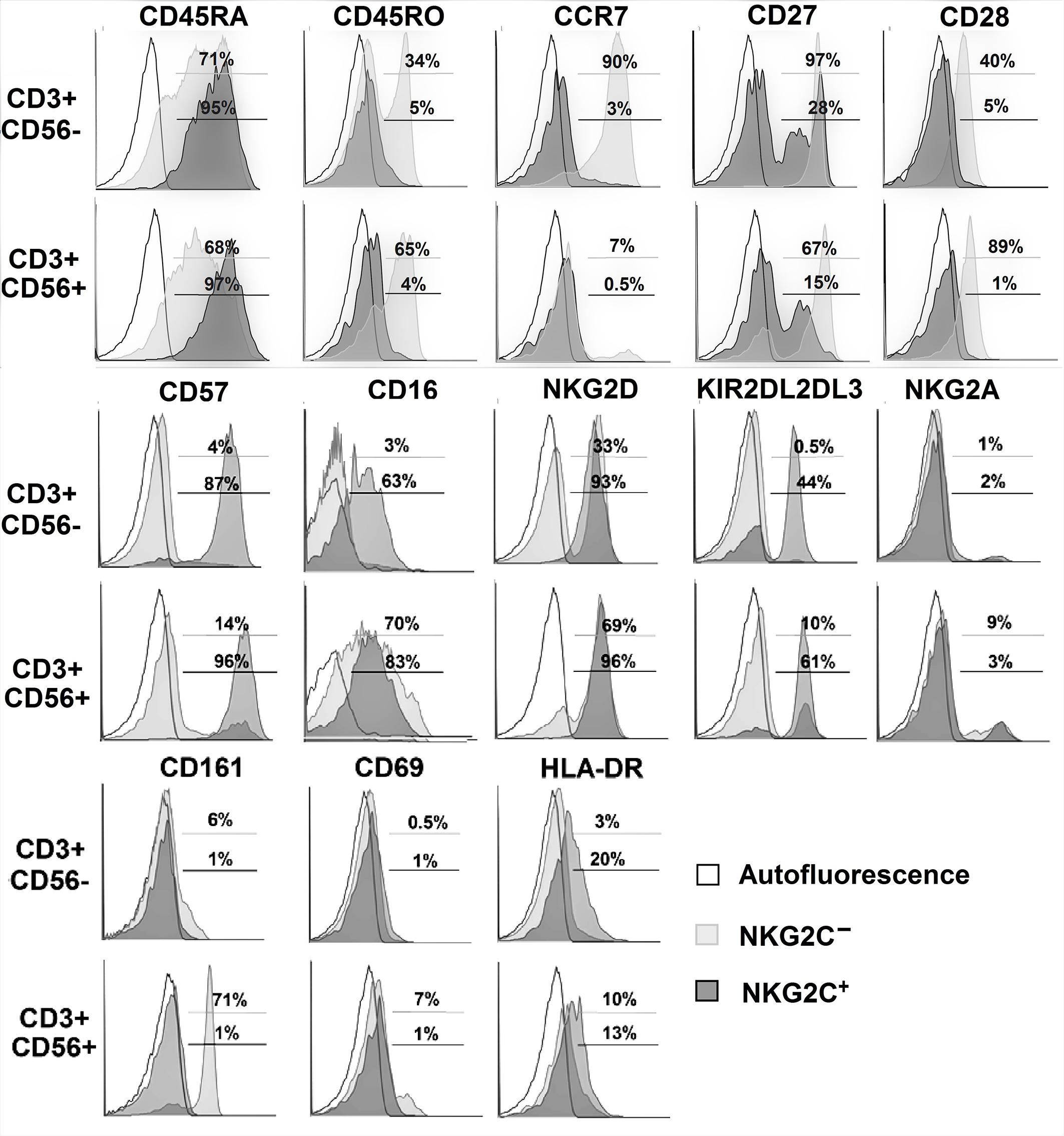

Supplement: Supplementary file 1 [file Image_1.tif]
